# Role of 2-[^18^F] Fluoro-2-Deoxy-D-Glucose-Positron Emission Tomography/Computed Tomography in the Post-Therapy Surveillance of Breast Cancer

**DOI:** 10.1371/journal.pone.0115127

**Published:** 2014-12-17

**Authors:** Hong-Tai Chang, Chin Hu, Yu-Li Chiu, Nan-Jing Peng, Ren-Shyan Liu

**Affiliations:** 1 Department of Surgery, Kaohsiung Veterans General Hospital, Kaohsiung, Taiwan; 2 Department of Nuclear Medicine, Kaohsiung Veterans General Hospital, Kaohsiung, Taiwan; 3 National Yang-Ming University, School of Medicine, Taipei, Taiwan; 4 Department of Nuclear Medicine, Taipei Veterans General Hospital, Taipei, Taiwan; Stanford University School of Medicine, United States of America

## Abstract

**Purpose:**

To evaluate the usefulness of 2-[^18^F] fluoro-2-deoxy-D-glucose-positron emission tomography/computed tomography (FDG-PET/CT) in the early detection of breast cancer tumor recurrences and its role in post-therapy surveillance.

**Methods:**

FDG-PET/CT was performed on patients with increased serum CA 15-3 levels and/or clinical/radiologic suspicion of recurrence. A group of asymptomatic patients who underwent FDG-PET/CT in the post-therapy surveillance of breast cancer served as the controls. The results were analyzed based on the patients' histological data, other imaging modalities and/or clinical follow-up. Recurrence was defined as evidence of recurrent lesions within 12 months of the FDG-PET/CT scan.

**Results:**

Based on elevated serum CA15-3 levels (n = 31) and clinical/radiologic suspicion (n = 40), 71 scans were performed due to suspected recurrence, whereas 69 scans were performed for asymptomatic follow-up. The sensitivity and specificity of FDG-PET/CT were 87.5% and 87.1% in the patients with suspected recurrence and 77.8% and 91.7% in the asymptomatic patients. The positive predictive value in the patients with suspected recurrence (mainly due to elevated serum CA 15-3 levels) was higher than that in asymptomatic patients (*P* = 0.013). Recurrences were proven in 56.3% (40/71) of the patients with suspected recurrence and in 13% (9/69) of the asymptomatic patients (*P*<0.001). FDG-PET/CT resulted in changes in the planned management in 49.3% (35/71) of the patients with suspected recurrence and 10.1% (7/69) of the asymptomatic patients (*P*<0.001). After follow-up, 77.5% (55/71) of the patients with suspicious recurrences and 97.1% (67/69) of the asymptomatic patients were surviving at the end of the study (*P*<0.001).

**Conclusions:**

FDG-PET/CT was able to detect recurrence, and the results altered the intended patient management in the post-therapy surveillance of breast cancer. FDG-PET/CT should be used as a priority in patients with increased serum CA 15-3 levels, or with clinical/radiologic suspicion of recurrence, and might be useful for asymptomatic patients.

## Introduction

Increasingly, breast cancer is becoming a leading cause of cancer-related mortality in the Western and Eastern worlds [1], [2]. The incidence of breast cancer has increased 4-fold in the past twenty years. In Taiwan, it has become the most common female cancer, and the fourth cause of cancer deaths in women [2]. Depending on the extent of the disease, up to 35% of patients who receive full treatment (surgery and others) will ultimately develop local recurrence or secondary tumor dissemination to distant organs [3].

Serum CA 15-3 has been used as a tumor marker for detecting asymptomatic or early recurrences of breast cancer that might be amenable to curative therapy. Although increased serum CA 15-3 levels precede the symptoms of metastasis by a mean time of 2-9 months, current international guidelines do not recommend its routine use for screening for metastases because of its moderate sensitivity and the absence of clinical impact [4]–[6]. Furthermore, additional imaging examinations are required to detect the exact location and extension of recurrent lesions.

Positron emission tomography with the glucose analog tracer 2-[^18^F]fluoro-2-deoxy-D-glucose (FDG-PET) is an imaging method that is based on the increase in glucose metabolism in malignant tumors. In breast cancer patients, FDG-PET has been reported to be useful in evaluating the initial tumor (including locoregional or distant staging), in evaluating treatment response, and in assessing recurrent disease [7]–[10]. A meta-analysis of 16 clinical reports that included 808 patients determined that the sensitivity and specificity of FDG-PET in detecting breast cancer recurrences and metastases was 92.7% and 81.6%, respectively [11]. Currently, integrated PET/CT has emerged as a promising imaging modality that is being applied more routinely in clinical situations. PET/CT evaluates both the metabolic activity and anatomy at the same location in the body, and is useful in accurately staging metastatic disease, assessing response to systemic treatment and clarifying equivocation with other imaging modalities [12]. A systematic review of the diagnosis of breast cancer recurrence showed that PET/CT has significantly higher sensitivity than PET (96% vs. 85%, *P* = 0.006) and conventional imaging tests (95% vs. 80%, *P* = 0.015), and higher specificity than PET (89% vs. 82%) and conventional imaging tests (89% vs. 77%). Changes in the management of the study patients ranged from 11% to 74% [13].

Early detection of recurrent cancer in a suspected or even asymptomatic patient provides more treatment options and offers a better prognosis and a good outcome. PET/CT scans are more sensitive than conventional imaging, and the detection of unsuspected lesions often alters the therapeutic plans. However, there is no consensus on whether PET/CT scans should be used as a standard component of the restaging evaluation in women with suspected recurrence or an asymptomatic patient. The aim of this study was to evaluate the role of FDG-PET/CT in the post-therapy surveillance of breast cancer.

## Materials and Methods

### Patients

We retrospectively collected data from patients with prior histories of breast cancer and complete responses to treatment (i.e., primary surgery and/or chemotherapy). The patients, who underwent FDG-PET/CT scans from June 2006 to Jan. 2012, were divided into 2 groups as follows: patients with elevated serum CA 15-3 levels>31.3 U/ml [14] (Group 1) or patients with clinical/radiologic suspicions recurrences without increased serum CA 15-3 levels (Group 2). Data from another group of patients who underwent FDG-PET/CT for asymptomatic post-therapy surveillance of recurrent breast cancer from our institution were also collected for comparison. The exclusion criteria for this study included stage IV of breast cancer at the time of initial diagnosis, vital sign instability, severe diabetes, severe illness, confirmed existence of 1 or more additional tumors and an inability to remain supine for 30 minutes. Finally, 140 FDG-PET/CT consecutive studies of patients with prior breast cancer after complete therapy were obtained. According to the study application forms, 71 studies were referred due to suspected recurrence, the indications of which included increased CA 15-3 levels (n = 31), suspicious or indeterminate lesion on sonography (n = 14), CT scans (n = 7), whole body bone scan (n = 3), mammography (n = 3) and chest film (n = 1), palpable breast or axillary nodules (n = 9), clinical symptoms of recurrence (n = 2), and the elevation of other serum tumor markers (n = 1). The detailed patient characteristics are shown in Table 1. The American Joint Committee on Cancer (AJCC) classification was used for the initial breast cancer staging. This study was approved by the Kaohsiung Veterans General Hospital institutional review board. The written or verbal informed consent to participate in this study was waived by the hospital ethics committee because of the retrospective nature of the study.

**Table 1:** Patient Characteristics.

	Suspected recurrent (n = 71)	Asymptomatic (n = 69)	*P*
Median age, years (range)	51 (33–84)	49(29–81)	0.239
Location of tumors			
Left	34	28	0.384
Right	35	41	0.384
Left & Right	2	0	
Tumor, type			
Invasive duct carcinoma	64	62	0.955
Invasive lobular carcinoma	5	4	
Papillary	1	1	
Metaplastic carcinoma	0	2	
Atypical medullary carcinoma	1	0	
Stage at diagnosis			
0	1	1	
I	13	19	
II	34	30	
III	23	19	
IV	0	0	

### FDG PET/CT imaging

The patients fasted for at least 6 hours prior to the FDG PET/CT imaging. An intravenous catheter was placed for radiopharmaceutical administration, and the patient's blood glucose levels were measured prior to injecting the tracer. All patients had blood glucose levels <150 mg/dl at the time of injection. Each patient received 370–555 MBq of ^18^F-FDG based on body weight (7.03 MBq/kg). After the tracer injection, the patients rested for 1 hour on a comfortable bed in a dark room. Whole-body FDG-PET/CT imaging (Discovery ST-16; GE Healthcare, Milwaukee, WI, USA) was performed from the head to the upper thigh with the patients in a supine position. A delayed image with or without the use of diuretics was obtained when necessary. CT scanning was performed prior to acquiring the PET data. The following parameters were used: 0.6 seconds per rotation: 120 kV, 100 mA, and 3.75-mm-thick slices. After performing plain CT, PET images of the same areas were acquired in a two-dimensional mode, and 4 minutes of data were collected per bed position. Attenuation-corrected PET images were reconstructed with an ordered subset expectation maximization iterative reconstructed algorithm. The 3.75-mm thick transaxial CT images were reconstructed at 3.27-mm intervals for fusion with the PET images. PET, CT, and fused PET/CT images were generated on a Xeleris image display and processing platform (GE Healthcare) for review on a computer workstation.

### Image analysis

The PET, CT, and fused PET/CT images were interpreted by two qualified nuclear medicine physicians who were allowed to manipulate the image contrast, image intensities, and three-dimensional images on a computer screen. The final diagnoses were made by consensus. The physicians were not blinded to the reasons for the examinations or the outcomes at the times of the image analysis. Because this was a retrospective study, the reasons for the examination were described on the application form. Each study was regarded as an independent event, and prior imaging, including prior PET/CT and contrast-enhanced CT scans, was available at the time of review to enable the fullest analysis. Both physicians reviewed the data independently before reaching consensus. Any increases in the FDG uptake were compared with the corresponding anatomical findings on the CT scan. For lesions that demonstrated abnormal FDG uptake, the physicians outlined the region of interest that indicated the area with the greatest amount of uptake. A standardized uptake value (SUV) was determined semi-automatically using the SUV tools that are available in the Xeleris software (SUV = activity in the region of interest [Bq/g]/[injected dose (Bq)/body weight (g)]). Two-dimensional regions of interest were drawn around the tumor on each transaxial slice that contained tumor tissue. A single-pixel SUVmax was determined for each region, and the slice that demonstrated the highest SUV was designated as the SUVmax for the entire tumor.

### Statistical analysis

The results were analyzed on a pathological basis (when histological sampling was possible), on other imaging modalities, and/or on clinical follow-up evaluations. Recurrent breast cancer was defined as the detection of recurrent lesions within 12 months of the FDG-PET/CT scan. A chi-squared test was used to determine the significance of the differences between the two groups with respect to detecting recurrent disease and patient outcomes. Differences were considered to be significant if *P*<0.05. All calculations were performed with SPSS version 12.0 (SPSS, Chicago, IL, USA).

## Results

The outcomes of the FDG-PET/CT studies in patients with suspected recurrence and asymptomatic patients are shown in Table 2. Serum CA 15-3 levels were obtained within 3 months of the FDG-PET/CT studies. Among the 140 FDG-PET/CT studies, recurrences were proven in 49, including 40 of 71 (56.3%) in patients with suspected recurrence and 9 of 69 (13%) in asymptomatic patients, respectively (*P*<0.001). Of the recurrences, 16 (32.7%) were proven on a histological basis, and the others were proven by other imaging modalities as disease progression or responses to therapy during follow-up. After a median follow-up time of 32 months (ranged from 5 to 72 months) in the suspected recurrence group, 55 patients were surviving. In contrast, after a median follow-up time of 58 months (ranged from 4 to 73 months) in the asymptomatic group, 67 patients were surviving. There was a significant difference between these two groups (77.5% vs. 97.1%, *P*<0.001).

**Table 2:** The outcome of FDG-PET/CT studies in patients with suspected recurrence and in asymptomatic patients.

	Suspected recurrent (n = 71)	Asymptomatic (n = 69)	*P*
Median interval to last surgery, months (range)	37 (5–204)	42 (7–168)	0.16
Median CA 15-3 level, ng/ml (range)	19.6 (2.1–1329)	11.4 (4.2–31.1)	0.09
Median follow-up time, months (range)	32 (5–72)	58 (4–73)	<0.001
Recurrence, N (% over studies)	40 (56.3%)	9 (13%)	<0.001
Median tumor SUVmax (range)	5.7 (1.1–23.2)	3.8 (2.3–7.7)	<0.001
Distant metastasis, N (% of recurrences)	21 (52.5%)	3 (33.3%)	0.3
Time to recurrence, months (range)	37 (7–204)	41 (28–172)	0.25
Time from recurrence to therapy, months (range)	1 (1–10)	3 (1–11)	0.14
Histology, N (% of studies)	18 (25.4%)	6 (8.7%)	0.009
Death, N (% of studies)	16 (22.5%)	2 (2.9%)	<0.001

### Diagnostic value of FDG-PET/CT

**Figure 1:**
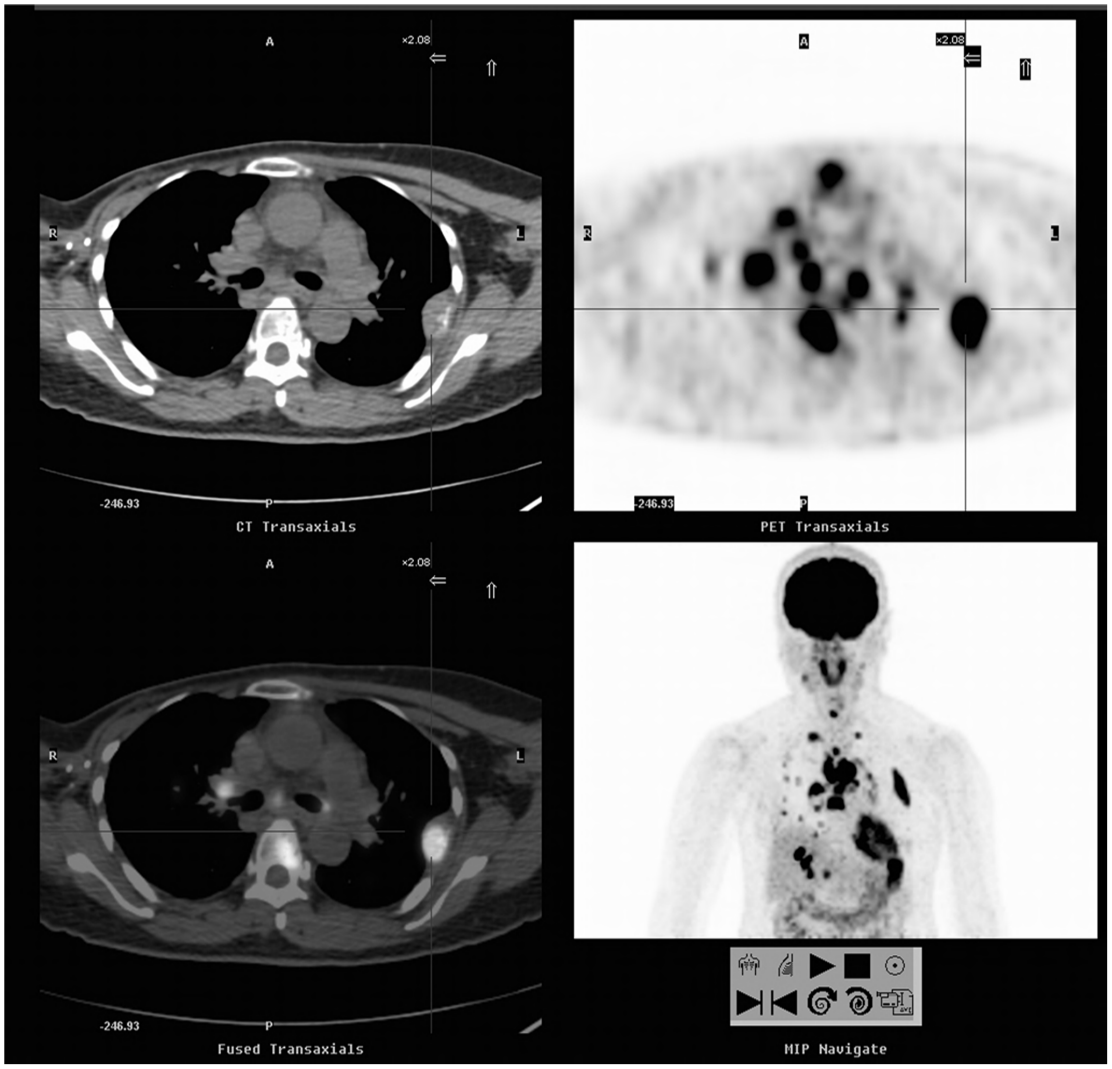
A 55-year-old woman with a history of breast cancer (invasive ductal carcinoma of the right breast, stage I) underwent a FDG-PET/CT scan due to elevated serum CA15-3 level of 44.7 U/ml. FDG-PET/CT showed local recurrence in right anterior chest wall, extensive lymphatic invasion, and multiple metastases to liver, lungs and bones (SUVmax = 17.5, cross cursor). The patient's disease progressed rapidly, and she expired 8 months later. FDG-PET/CT, 2-[^18^F]fluoro-2-deoxy-Dglucose-positron emission tomography/computed tomography; SUV, standardized uptake value.

The use of FDG-PET/CT detected 42 recurrent cancers (Table 3) and 9 false-positives. Of the former, distant metastasis was found in 60% (21/35) of the patients with suspected recurrence (Fig. 1) and in 42.9% (3/7) of the asymptomatic patients. The median tumor SUVmax in patients with suspected recurrence and asymptomatic patients was 5.7 (ranged 1.1 to 23.2) and 3.8 (ranged 2.3 to 7.7), respectively (*P*<0.001). Two chest wall lesions less than 1 cm (0.7 cm and 0.8 cm, respectively) with SUVmax of 1.4 and 2.3 and another two abnormalities on a CT (internal mammary chain and pleura) with SUVmax of both 2.3 were interpreted as being positive for recurrent disease. Of the latter group, 4 cases were proven with histological analysis, including 2 cases of second primary breast cancers with different histopathologic subtypes and 2 cases of fat necrosis over prior operation sites. The remaining cases were confirmed as benign in follow-up studies and included 2 cases of pulmonary tuberculosis, 1 reactive node case and 2 cases with unknown causes of increased FDG uptake in the liver and bone. Seven false-negative diagnoses were noted, 4 of which identified malignancies (based on histology) of liver metastasis, recurrent breast lesion, liver metastasis and malignant pleural effusion at 1, 3, 9 and 11 months, respectively, after the PET/CT scan. The recurrent breast lesion, a 0.5 cm nodule with a SUVmax of 1.1, was interpreted as being a benign lesion. An excisional biopsy of the nodule was performed, and the pathological analysis revealed metastasis. The other 3 cases recurred within 12 months, with disease progression (n = 2) and response to therapy (n = 1) during follow-up. In the remaining patients, no recurrent disease was detected during the follow-up period of at least 12 months, which included close surveillance, the periodic evaluation of serum CA 15-3 levels and other imaging modalities.

**Table 3:** Detailed information about the patients with recurrent breast cancers and positive FDG-PET/CT findings.

Case	Age (year)	CA 15-3 (ng/ml)	Stage	First treatment	FDG-PET/CT findings	SUVmax
1	55	44.7	I	BCT	Chest wall, LN, lung, liver, bone.	17.5
2	46	36	II	MRM	Mediastinal LN	4.7
3	60	40	III	MRM	Bone	6.1
4	55	98	II	MRM	Internal mammary chain	2.3
5	46	145.5	II	MRM	Chest wall, LN, bone	15.3
6	33	33.3	I	SSM+TRAM	Lung, LN	13.6
7	34	35.8	II	MRM	LN	3.9
8	36	33.1	II	MRM	Chest wall (a 0.7 cm nodule)	1.4
9	75	33	III	MRM	Bone	4.8
10	57	60.4	III	MRM	Liver	5.7
11	47	1329	II	MRM	Lung, bone	14.8
12	57	32.3	II	MRM	Bone	7.6
13	55	52.4	III	MRM	Bone	5.6
14	38	38.4	II	SSM+TRAM	Chest wall, LN	4.3
15	39	55.8	III	SSM+TRAM	Chest wall, LN, liver	17.5
16	68	43.4	III	MRM	Bone	3.5
17	77	31.4	III	MRM	Chest wall, LN, lung	4.1
18	45	34.3	II	MRM	Chest wall, LN, bone	11.2
19	50	27	II	MRM	Chest wall, LN, bone	7.7
20	57	18.9	III	MRM	Chest wall, LN	20.6
21	84	18.9	II	MRM	LN, lung, adrenal gland	6.8
22	70	19.6	II	MRM	LN, lung, liver	10.8
23	47	16.1	II	MRM	Chest wall, LN	8.2
24	43	21	I	Lumpectomy+ALND	Chest wall	23.2
25	38	12.7	II	SSM+TRAM	LN (a 1.1 cm axillary node)	4.7
26	43	2.1	II	SSM+TRAM	LN, lung, bone	8.1
27	48	10.1	III	MRM	Liver	2.9
28	40	7.9	I	BCT	Chest wall	3.8
29	68	14.1	II	MRM	Chest wall (a 0.8 cm nodule)	2.3
30	63	7.1	II	MRM	LN	18.2
31	76	10.6	II	MRM	Chest wall, LN	12.1
32	60	19.5	I	MRM	LN, lung	9.9
33	60	7.5	II	Partial mastectomy	LN	9.1
34	62	9.2	II	BCT	Chest wall, LN	4
35	58	16.6	II	MRM	Lung	2.6
36	42	8.4	II	MRM	LN	3.2
37	45	20.9	I	Partial mastectomy+ALND	Pleura	2.3
38	49	23.6	0	Excision	LN	3.4
39	55	10.5	III	MRM	LN, bone	4.1
40	81	23.1	III	MRM	Lung	3.3
41	46	5.8	III	SSM+TRAM	LN	7.7
42	59	21.8	I	BCT	LN, bone, lung	6.8

ALND: axillary lymph node dissection; BCT: breast conserving therapy; LN, lymph node; MRM: modified radical mastectomy; SSM+TRAM: skin-sparing mastectomy and transverse rectus abdominus myocutaneous; SUV, standardized uptake value.

Accordingly, the sensitivity, specificity, positive predictive value (PPV), negative predictive value (NPV) and accuracy of FDG-PET/CT were as follows: overall, 85.7%, 90.1%, 82.4%, 92.1% and 88.6%, respectively; in patients with suspected recurrence, 87.5%, 87.1%, 89.7%, 84.4% and 87.3%, respectively; and in asymptomatic patients, 77.8%, 91.7%, 58.3%, 96.5% and 89.9%, respectively (Table 4).

**Table 4:** Effectiveness of CA 15-3 and FDG-PET/CT in detecting recurrent breast cancer in patients with suspected recurrence and in asymptomatic patients.

	TP	FP	TN	FN	Sensitivity	Specificity	PPV	NPV	Accuracy
Serum CA 15-3									
Suspected recurrence (n = 71)	19	12	19	21	47.5%*	61.3%†	61.3%*	47.5%*	53.5%*
Group 1 (n = 31)	19	12	0	0					
Group 2 (n = 40)	0	0	19	21					
Asymptomatic (n = 69)	0	0	60	9					
Total (n = 140)	19	12	79	30	38.8%	86.8%	61.3%	72.5%	70%
FDG-PET/CT									
Suspected recurrence (n = 71)	35	4	27	5	87.5%*	87.1%†	89.7%* §	84.4%*	87.3%*
Group 1 (n = 31)	18	1	11	1	94.7%	91.7%	94.7%∣	91.7%	93.5%
Group 2 (n = 40)	17	3	16	4	80.9%	84.2%	85%	80%	82.5%
Asymptomatic (n = 69)	7	5	55	2	77.8%	91.7%	58.3%§ ^∣^	96.5%	89.9%
Total (n = 140)	42	9	82	7	85.7%	90.1%	82.4%	92.1%	88.6%

FN, false negative; FP, false positive; PPV, positive predictive value, NPV, negative predictive value, TN, true negative; TP, true positive; Group 1, patients with elevated serum CA 15-3 levels>31.3 U/ml; Group 2, patients with clinical/radiologic suspicions of recurrences without serum CA 15-3 level increases;

*, *P*<0.01 FDG-PET/CT vs. serum CA 15-3;

† , *P* = 0.04 FDG-PET/CT vs. serum CA 15-3;

§ , *P* = 0.013 suspected recurrence vs. asymptomatic;

∣ , *P* = 0.012 Group 1 vs. asymptomatic.

### Diagnostic value of serum CA 15-3

We compared the effectiveness of serum CA 15-3 levels with that of FDG-PET/CT in detecting recurrent cancer. In patients with suspected recurrence, FDG-PET/CT demonstrated sensitivity, specificity, PPV, NPV and accuracy of 87.5%, 87.1%, 89.7%, 84.4% and 87.3%, respectively, which were significantly higher than the 47.5%, 61.3%, 61.3%, 47.5% and 53.5%, respectively, which were demonstrated by elevated serum CA 15-3 levels. FDG-PET/CT also showed higher sensitivity, specificity, PPV, NPV and accuracy of 94.7%, 91.7%, 94.7%, 91.7% and 93.5%, respectively, in patients with elevated serum CA 15-3 levels (Group 1) compared to 80.9%, 84.2%, 85%, 80% and 82.5%, respectively, in patients without elevated serum CA 15-3 levels (Group 2). However, these differences were not significantly different (Table 4). However, the PPV in the patients with suspected recurrence and elevated serum CA 15-3 levels was higher than that in asymptomatic patients (*P* = 0.013 and 0.012, respectively).

### Impact of FDG-PET/CT on patient management

**Figure 2:**
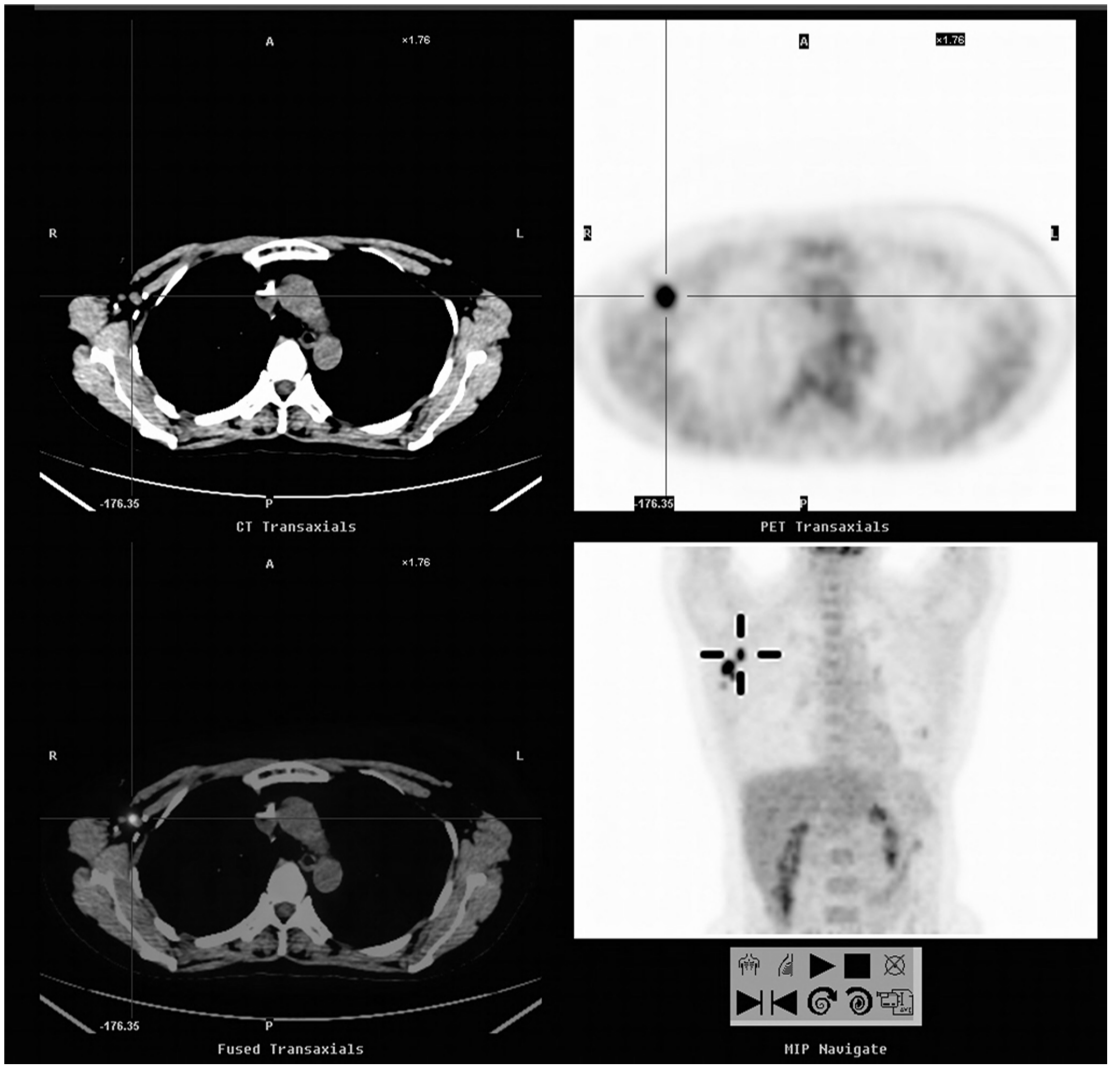
A 46-year-old woman with a history of breast cancer (invasive ductal carcinoma of the right breast, stage IIIA) underwent a FDG-PET/CT scan for asymptomatic follow-up. FDG-PET/CT showed glucose hypermetabolism in the right axillary nodes (SUVmax = 7.7, cross cursor). The pathological analysis confirmed the presence of metastatic lymph nodes. The patient's serum CA 15-3 remained within normal ranges throughout the course. The patient remained on clinical follow-up at the end of the study.

In patients with FDG-PET/CT evidence of disease recurrence (n = 42), 35 (83.3%) underwent chemotherapy alone, 4 (9.5%) underwent combined therapy with surgery, and 3 (7.1%) underwent radiotherapy. FDG-PET/CT changed the planned management in 30% (42/140) of the patients, including 49.3% (35/71) of patients with suspected recurrence and 10.1% (7/69) of asymptomatic patients (*P*<0.001) (Fig. 2). In patients with suspected recurrence, the therapeutic management was changed in 49.3% (35/71) based on positive FDG-PET/CT scans, and for 26.8% (19/71) of the patients with increased serum CA 15-3 levels (*P* = 0.006).

## Discussion

FDG-PET/CT is increasingly being recognized as a powerful tool for evaluating patients with various malignant tumors. The advantages of FDG-PET/CT in detecting recurrent breast cancer include the high sensitivity, reasonable specificity and the ability to screen the entire body in one imaging session. Previous studies have included different proportions of recurrent patients ranging from 68–93% [7]–[10], [15], and the incidence of distant metastasis ranged from 70–81% [10], [15]. The proportion of recurrence (35%, 49/140) and the incidence of distant metastasis (49%, 24/49) in our patients were lower than those reported in past studies. This difference might be because, having shown significant benefit in recent years, more aggressive breast cancer treatments are being used [10]–[11], [16]. The early detection and accurate restaging of recurrent breast cancer is important for selecting the appropriate therapeutic strategy, which may offer a better prognosis and a good outcome. In the presented study, recurrences were diagnosed in 56.3% (40/71) of patients with suspected recurrence and also in 13% (9/69) of asymptomatic patients. FDG-PET/CT resulted in changes in 49.3% (35/71) of patients with suspected recurrence and also in 10.1% (7/69) of asymptomatic patients.

Blood tumor markers are widely used to follow patients who are being treated for malignant tumors. However, the low incidence of elevated serum CA 15-3 concentrations in early-stage cancer suggests that this marker lacks the necessary sensitivity for detecting early metastatic disease. In the present study, 38.8% (19/49) of recurrent patients had elevated serum CA 15-3 levels before or at the time of recurrence; this result was aligned with prior studies that reported the sensitivity of serum CA 15-3 for detecting recurrent breast cancer ranged from 35.3% to 50% [16]–[18]. The American Society of Clinical Oncology (ASCO) recommendations for the use of tumor markers does not support the use of serum CA 15-3 to monitor disease recurrence during follow-up for treated breast cancer [6]. Conversely, FDG-PET/CT is useful in detecting recurrent breast cancer. In our study, PET/CT detected 42 of 49 recurrent cancers, with a sensitivity and specificity of 85.7% and 90.1%, respectively, which aligns with prior studies, in which the sensitivity and specificity ranged from 81% to 96% and 52% to 89%, respectively [13], [15]–[19]. In this study FDG-PET/CT showed a higher sensitivity and specificity of 94.7% and 91.7%, respectively, in patients with elevated serum CA15-3 level compared to other groups of patients without elevated serum CA15-3 level, although these results were not statistically significant. However, the PPV in the patients with increased serum CA 15-3 levels was higher than that in asymptomatic patients (*P* = 0.012). In some countries, serum CA 15-3 still plays a role in patient follow-up for monitoring distant recurrence [20]–[22]. It also, serves as a prognostic factor [4]–[5], and has been used as an inclusion criterion for further imaging examinations in cases of suspected recurrence [7]–[13], [15]–[16], [19]. Grassetto G et al. conducted a multicenter study that suggested FDG-PET/CT plays a potential role in asymptomatic patients with rising serum CA 15-3 and negative conventional imaging [23]. In their study, the sensitivity of serum CA 15-3 was similar to that in our patient group (45% vs. 47.5%). FDG-PET/CT could detect the smallest lesion (0.7 cm), as was the case in this study. However, more cases with a solitary lesion amenable to radical therapy were present in their study (57.5%, 23/40), which might be due to timely follow-up of serum CA 15-3 and the close monitoring of the high risk patient group and highly selected patients with negative conventional imaging. Our data are in agreement with a number of studies [7]–[13], [23], and suggest the timely detection of early recurrence such as by using tumor markers and FDG-PET/CT, has a major impact on therapy and survival.

Recently, published data from the National Oncologic PET Registry concerning patients with a variety of cancers have shown that the intended management changed for 36.5% of the patients following PET [24]. In a retrospective study of 125 patients with recurrent or metastatic breast cancer, the therapeutic plan was altered in 32% of the patients based on the FDG-PET findings [10]. In another study of 134 patients with suspicion of recurrence either clinically or on conventional imaging (suspected recurrence: [SR]) or with isolated increases in tumor marker levels (occult recurrence: [OR]), a 44% overall change in management, 43% in the SR group and 45% in the OR group were observed [7]. In the current study, FDG-PET/CT results changed the planned management in 30% of the patients, more so for patients with suspected recurrence than in asymptomatic patients (49.3% vs. 10.1%, *P*<0.001).

FDG-PET/CT is also helpful for selecting patients who might derive a significant survival benefit from optimal surgical strategy. Although there are still inherent false positive and false negative results, patients might also benefit from these false-positive findings. In our study, false positives resulted in surgical resection of 2 second primary cancers that were independent of prior breast cancer. An earlier recognition of synchronous cancers might allow more effective treatment options and improve patient survival rates. Further, two patients with pulmonary tuberculosis were appropriately treated. Four false-negatives, derived from the definition of recurrent lesions, were found within 12 months of the FDG-PET/CT. FDG-PET/CT were limited by the resolution and the inability to detect microscopic metastasis as FDG-PET/CT is relatively less sensitive in detecting lesions <1 cm in size [25]–[26]. In this study, we detected two chest wall lesions less than 1 cm (0.7 cm and 0.8 cm, respectively), whereas a 0.5 cm recurrent breast lesion was interpreted as being a benign lesion.

Our study had limitations. First, this study was retrospective and FDG-PET/CT was randomly applied by asymptomatic patients themselves. Therefore, this might have resulted in some bias. Second, only 24 of the 140 (17.1%) patients were proven based on histology, and the others were diagnosed via other imaging modalities and/or clinical follow-up. Third, the total case numbers were small. Further studies with a prospective trial looking at the specific question of recurrence in asymptomatic patients would be of interest.

## Conclusions

FDG-PET/CT scans are useful in the early diagnosis of recurrent breast cancer and thus might improve the survival rates of these patients. In this study, FDG-PET/CT had high overall sensitivity and specificity for detecting recurrent breast cancer and higher PPV in the patients with increased serum CA 15-3 levels than that in the asymptomatic patients. Patients with suspected recurrence had a much greater proportion of recurrences, a greater incidence of distant metastasis and higher mortality rates than asymptomatic patients. Therefore, FDG-PET/CT should be used as a priority in patients with increased serum CA 15-3 levels, or with clinical/radiologic suspicion of recurrence, and might be useful for asymptomatic patients.
